# Editorial: Neural network models in autonomous robotics

**DOI:** 10.3389/fnbot.2025.1587137

**Published:** 2025-04-08

**Authors:** Long Cheng, Ying Mao, Tomas Ward

**Affiliations:** ^1^School of Control and Computer Engineering, North China Electric Power University, Beijing, China; ^2^Department of Computer and Information Science, Fordham University, New York City, NY, United States; ^3^Insight SFI Research Centre for Data Analytics, Dublin City University, Dublin, Ireland

**Keywords:** neural network models, autonomous robotics, energy efficiency, multi-modal sensory data, human-robot collaboration

## 1 Introduction

The integration of neural network models in autonomous robotics represents a monumental leap in artificial intelligence and robotics. These models, mirroring the human brain's complexity and efficiency, have catalyzed innovations in machine learning, fostering more adaptive, intelligent, and efficient robotic systems. Recent research in areas like deep learning, reinforcement learning, and neural network optimization has significantly advanced, yet challenges remain, especially in robotics' real-world application, energy efficiency, and operation in complex, unstructured environments.

This Research Topic aims to address these challenges by exploring innovative applications of neural network models in autonomous robotics. It seeks to highlight research that expands neural networks' role in enhancing robotic autonomy, decision-making, and adaptability. Contributions may include studies on energy-efficient neural network models for robotic systems, strategies to enhance robotic resilience in dynamic, real-world settings, or novel neural network architectures designed specifically for robotics, capable of processing diverse, multi-modal sensory inputs. The goal is to advance the field by presenting varied perspectives and methodologies that tackle current limitations and establish new benchmarks for future research.

## 2 Contents of the Research Topic

Following a rigorous review by at least two field experts, assessing originality, scientific rigor, methodological soundness, and potential impact, six papers were selected for inclusion. Together, they offer a rich exploration of algorithmic innovations in robotic navigation and sensing and learning-based perception and recognition in robotics, advancing the field with novel insights and solutions.

### 2.1 Navigation and planning

The article A Modified A* Algorithm Combining Remote Sensing Technique for Unmanned Surface Vehicle Sampling proposes a modified A* algorithm that integrates satellite-derived water quality data into path planning for unmanned surface vehicles. Balances path optimization with sample representativeness, validated in Chaohu Lake with improved Chlorophyll-a sampling accuracy (Wang et al.).

The article *A Survey of Decision-Making and Planning Methods for Self-Driving Vehicles* reviews knowledge-driven (rule-based, game theory) and data-driven (imitation/reinforcement learning) decision-making algorithms for autonomous vehicles. Highlights hybrid approaches, experimental platforms, and challenges for robust system design (Hu et al.).

The article *Robust Visual SLAM Algorithm Based on Target Detection and Clustering in Dynamic Scenarios* enhances visual SLAM by integrating improved YOLOv5 for dynamic object detection, filtering out moving features. Outperforms ORB-SLAM3 and DynaSLAM in dynamic environments with 85.7% higher accuracy and real-time efficiency (Gan et al.).

### 2.2 Learning-driven perception and recognition

The article *Latent Space Improved Masked Reconstruction Model for Human Skeleton-Based Action Recognition* combines masked autoencoders with VAE/VQVAE latent spaces to improve skeleton action recognition. Achieves higher classification accuracy and generalization on NTU-60/120 datasets, especially with limited labeled data (Chen et al.).

The article *ACA-Net: Adaptive Context-Aware Network for Basketball Action Recognition* proposes ACA-Net with temporal and spatial-channel modules for basketball action recognition in complex scenes. Achieves 89–92% accuracy on SpaceJam/Basketball-51 datasets, with applications in robotic game analysis (Zhang et al.).

The article *Learning-Based Object's Stiffness and Shape Estimation with Confidence Level in Multi-Fingered Grasping* develops a probabilistic neural network framework to estimate object stiffness, shape, and confidence levels (via uncertainty quantification) using proprioceptive signals. Enables reliable robotic manipulation in real time (Kutsuzawa et al.).

